# Supplementary material for: Microtubule Association of EML4–ALK V3 Is Key for the Elongated Cell Morphology and Enhanced Migration Observed in V3 Cells
Source: Cells. 2024 Nov 25;13(23):1954. doi: 10.3390/cells13231954 (PMC11639804; doi:10.3390/cells13231954)
Supplement: Supplementary file 1 [file cells-13-01954-s001.zip › SUPPLEMENTARY FIGURES.pdf]

SUPPLEMENTARY FIGURES

Figure S1. Cells expressing phosphomimetic EML4-ALK V3 mutants do not exhibit the elongated morphology or enhanced migratory phenotype. (A) Beas2B parental cells were transiently transfected with YFP-only, YFP: wild-type (WT) V3, YFP: double phosphomimetic (S144D/S146D) (DM) V3 mutant or YFP: triple phosphomimetic (S134D/S144D/S146D) (TM) V3 mutant, fixed and stained with antibodies against GFP (yellow) and α-tubulin (purple). Scale = 5 μm. (B) One-way ANOVA with Tukey's multiple comparisons tests were performed to measure colocalization (± SEM) between V3 and microtubules. Three biological repeats were performed (n = 28-29 cells in total per sample population) (colour-coded). (C) Lysates were also collected and prepared for Western blot analysis with antibodies against ALK, GFP and GAPDH. Molecular weights (kDa) indicated on the left. Three biological replicates were performed (n = 3). Blots and images are representative of all replicates. Uncropped blots are indicated in supplementary data. (D) Beas2B parental cells were transfected as before and fixed and stained with a GFP antibody. Scale = 50 μm. (E) The total cell length (μm ± SEM) and (F) protrusion length (μm ± SEM) were measured and one-way ANOVA with Tukey's multiple comparisons tests were performed. N = 70 cells in total per sample population (colour-coded). (G) Beas2B parental cells were transfected as before and subjected to live-cell imaging for 6 hours. Scale = 100 μm. Single cell tracking analysis was performed and the (H) total distance covered (μm ± SEM) and (I) average velocity (μm/min ± SEM) were measured. One-way ANOVA with Tukey's multiple comparisons tests were performed. **** $P < 0.0001$, ** $P = 0.0071$ or 0.0020 or 0.0034. N = 40 cells in total per sample population.

Figure S2. Generation and characterization of a stable U2OS: FKBP-V3 cell line. (A) Lysates were collected from 10 monoclonal populations of U2OS:FKBP-V3 cells and subjected for Western blot analysis with antibodies against ALK (FKBP-V3), mCherry (mCherry-FRB) and GAPDH. Uncropped blots are indicated in supplementary data. (B) Clones #3, #5, #6 and #9 were fixed and stained with antibodies against ALK and mCherry and images were captured using the 20X objective. Scale = 50 μm. Clones (C) #3 and (D) #5 were treated with 200 nM rapamycin for 2 hours and images captured using the 63X objective. Scale = 5 μm.

Figure S3. Control experiments to confirm possible NEK9-independent interaction of NEK7 with EML4-ALK V3. (A) Beas2B parental cells were treated with 200 nM rapamycin for 5 minutes before being fixed and stained with an antibody against NEK7 (green). Beas2B cells transfected with the FKBP-V3 construct were treated with 200 nM rapamycin before being fixed and (B) co-stained with antibodies against ALK and NEK7 or (C) ALK alone with secondary antibodies for both ALK and NEK7. Experiment was repeated three times (n = 3) and images are representative of all replicates. (D) Beas2B parental cells were transfected with the WT and TM V3 constructs before being fixed and co-stained with antibodies against NEK9 (yellow) and α-tubulin (purple). (E) One-way ANOVA with Tukey's multiple comparisons tests were performed to compare colocalization (± SEM). (F) Beas2B cells were transfected with the WT V3 construct before being fixed and co-stained with antibodies against NEK9 (purple) and ALK (yellow) and (G) unpaired t-tests were performed to compare the ALK: NEK9 co-localization (± SEM). (H) Beas2B cells were transfected with the FKBP-V3 construct before being fixed and co-stained with antibodies against NEK9 (rabbit antibody) (purple) and ALK (yellow) and (I) unpaired t-tests were performed to compare the ALK: NEK9 co-localization (± SEM). (A-H) Scale = 5 µm. (J) Doxycycline-inducible U2OS: aNEK9 cells were induced with 1 µg/ml doxycycline as indicated and lysates prepared for Western blot analysis with antibodies against *myc,* NEK9 (mouse antibody) and GAPDH or (K) fixed and stained with antibodies against NEK9 (mouse antibody) (green). Scale = 50 µm. Uncropped blots are indicated in supplementary data. (L) Unpaired t-tests were performed to compare the relative NEK9 intensity (± SEM) between non-induced and induced cells. **** $P < 0.0001$, ns = not significant. Experiments were performed in triplicates (n = 28-30 cells in total for each sample population) and each replicate is colour-coded where appropriate. All images and blots are representative of all replicates.

Figure S4. Use of doxycycline-inducible HeLa: aNEK7 or NEK7 KD cells to study the importance of NEK7 activation for the elongated morphology. (A) HeLa: aNEK7 or (B) NEK7 KD cells were induced with 1 µg/ml doxycycline for 0, 24, 48 and 72 hours before lysates were collected and prepared for Western blot analysis with antibodies against GFP and GAPDH. Molecular weights (kDa) are indicated on the left. Uncropped blots are indicated in supplementary data. (C) HeLa: aNEK7 transfected with the WT V3 construct were fixed and stained with α-tubulin and ALK. Three biological repeats were performed (n = 40 cells in total per sample population). (D) The total cell length (µm ± SEM) of individual cells was measured using both ALK and α-tubulin and compared using and unpaired t-test. ns = not significant. Two biological repeats were performed for all experiments (n = 20 cells

in total per sample population). Images and blots shown are representative of all replicates. Scale = 50 μm.

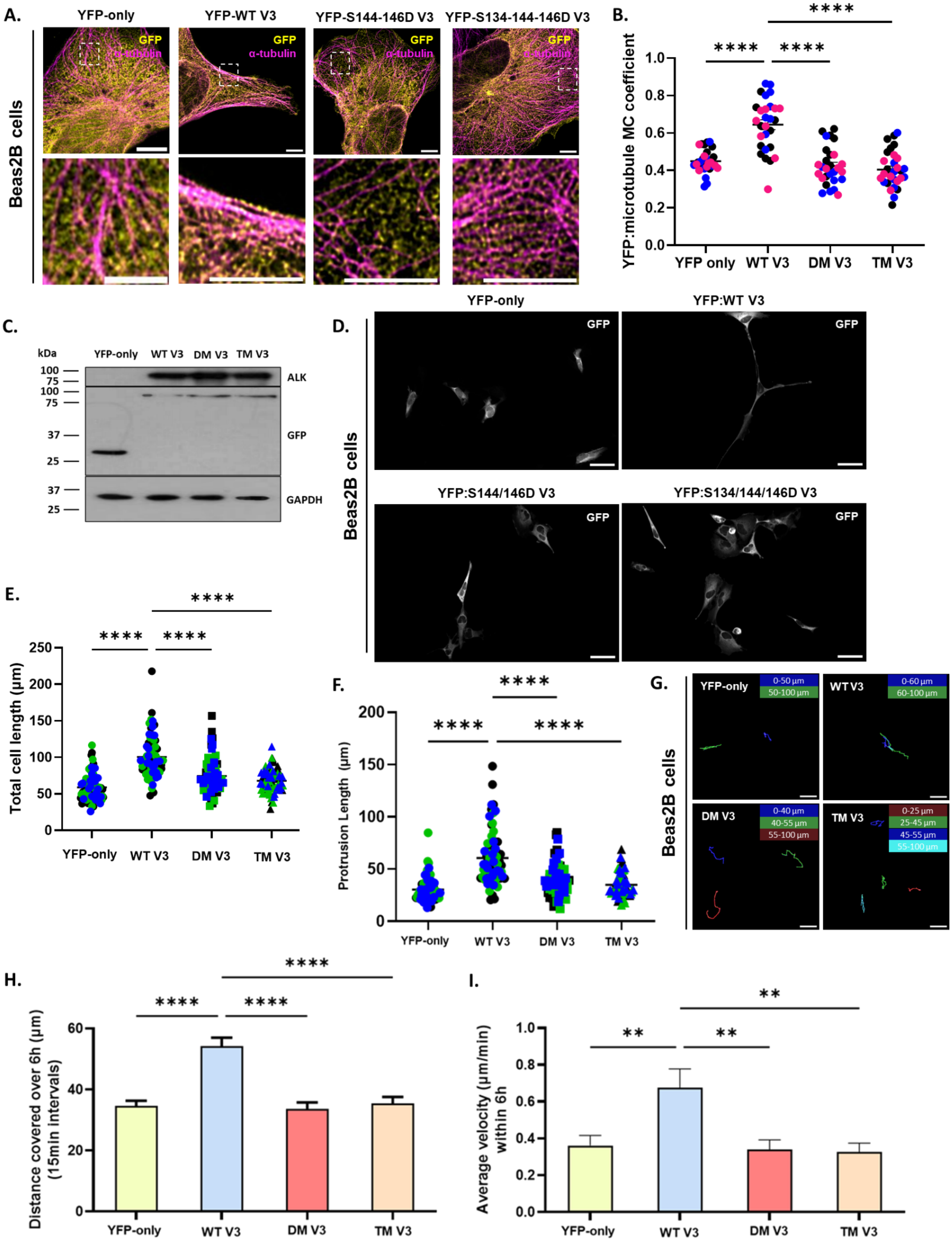
A.
YFP-only
YFP-WT V3
YFP-S144-146D V3
YFP-S134-144-146D V3
GFP
α-tubulin
Beas2B cells
B.
YFP:microtubule MC coefficient
YFP only
WT V3
DM V3
TM V3
****
C.
kDa
YFP-only
WT V3
DM V3
TM V3
ALK
GFP
GAPDH
D.
YFP-only
YFP:WT V3
YFP:S144/146D V3
YFP:S134/144/146D V3
E.
Total cell length (µm)
F.
Protrusion Length (µm)
G.
0-50 µm
50-100 µm
0-60 µm
60-100 µm
0-40 µm
40-55 µm
55-100 µm
0-25 µm
25-45 µm
45-55 µm
H.
Distance covered over 6h (µm)
(15min intervals)
I.
Average velocity (µm/min)
within 6h
**

Papageorgiou *et al*. (2024)_Figure S2

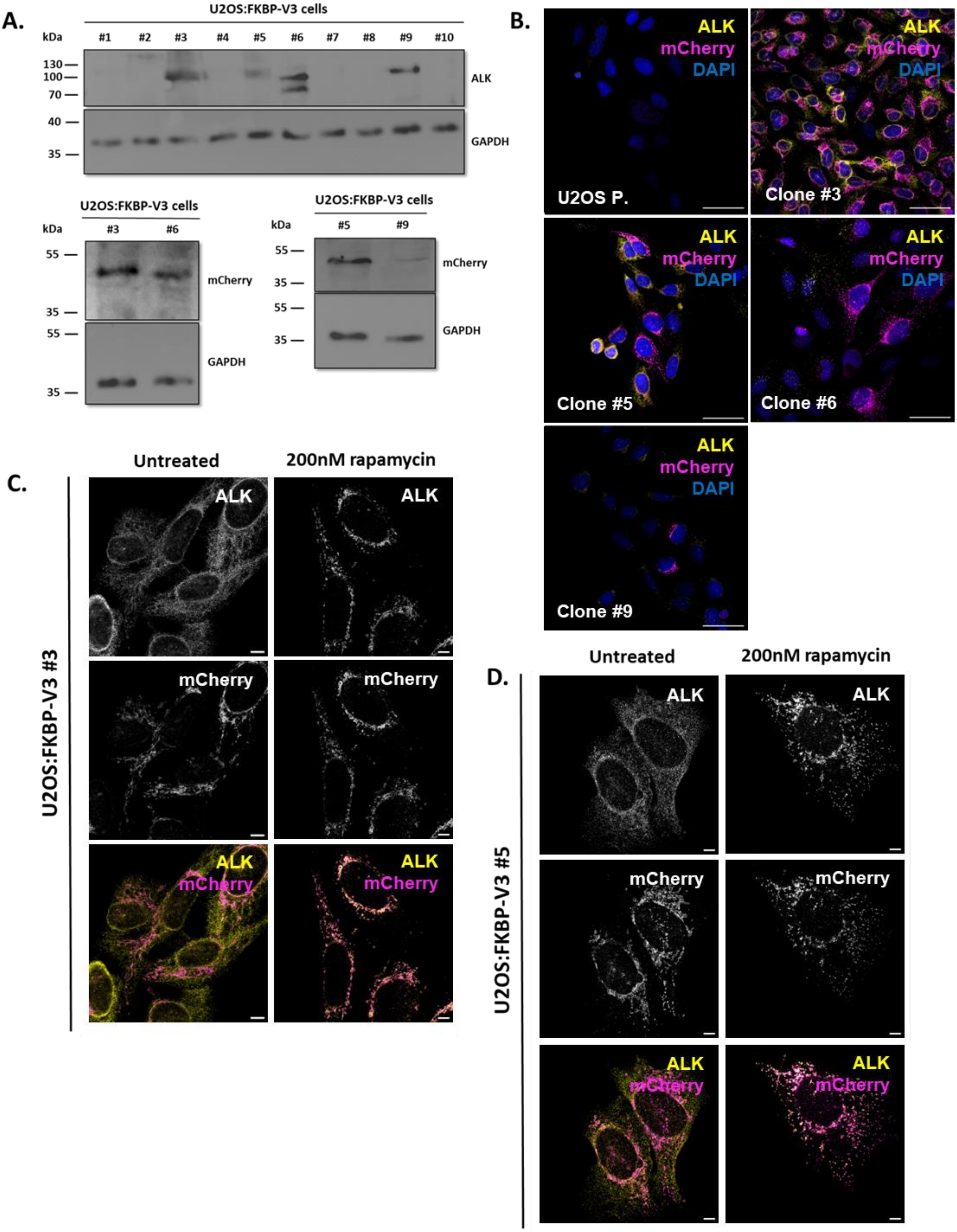

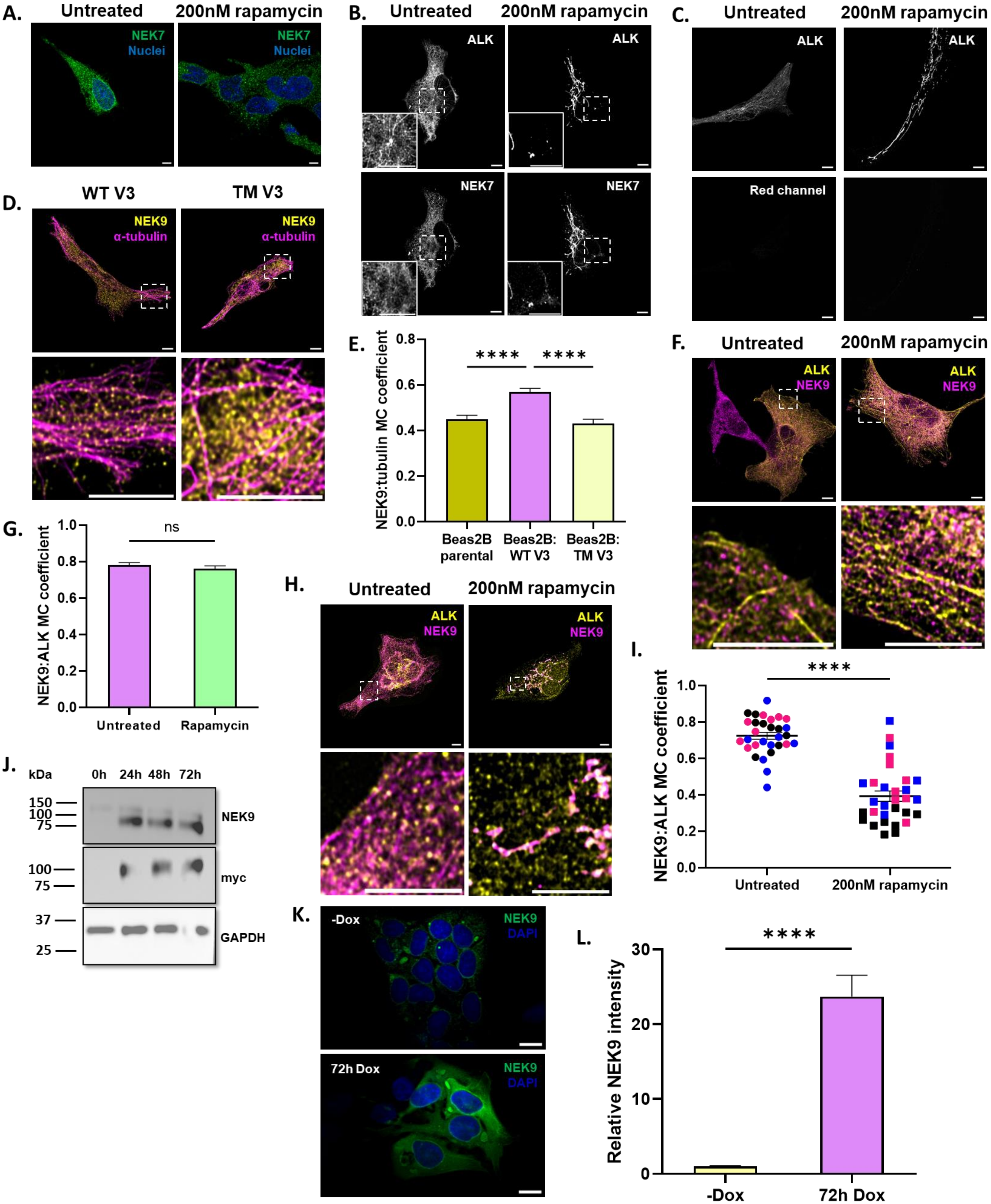
A.
Untreated
200nM rapamycin
NEK7
Nuclei
B.
ALK
NEK7
C.
Red channel
D.
WT V3
TM V3
NEK9
α-tubulin
E.
NEK9:tubulin MC coefficient
****
Beas2B parental
Beas2B: WT V3
Beas2B: TM V3
F.
ALK
NEK9
G.
NEK9:ALK MC coefficient
ns
Untreated
Rapamycin
H.
I.
J.
kDa
0h
24h
48h
72h
150
100
75
NEK9
myc
37
25
GAPDH
K.
-Dox
72h Dox
NEK9
DAPI
L.
Relative NEK9 intensity
-Dox
72h Dox

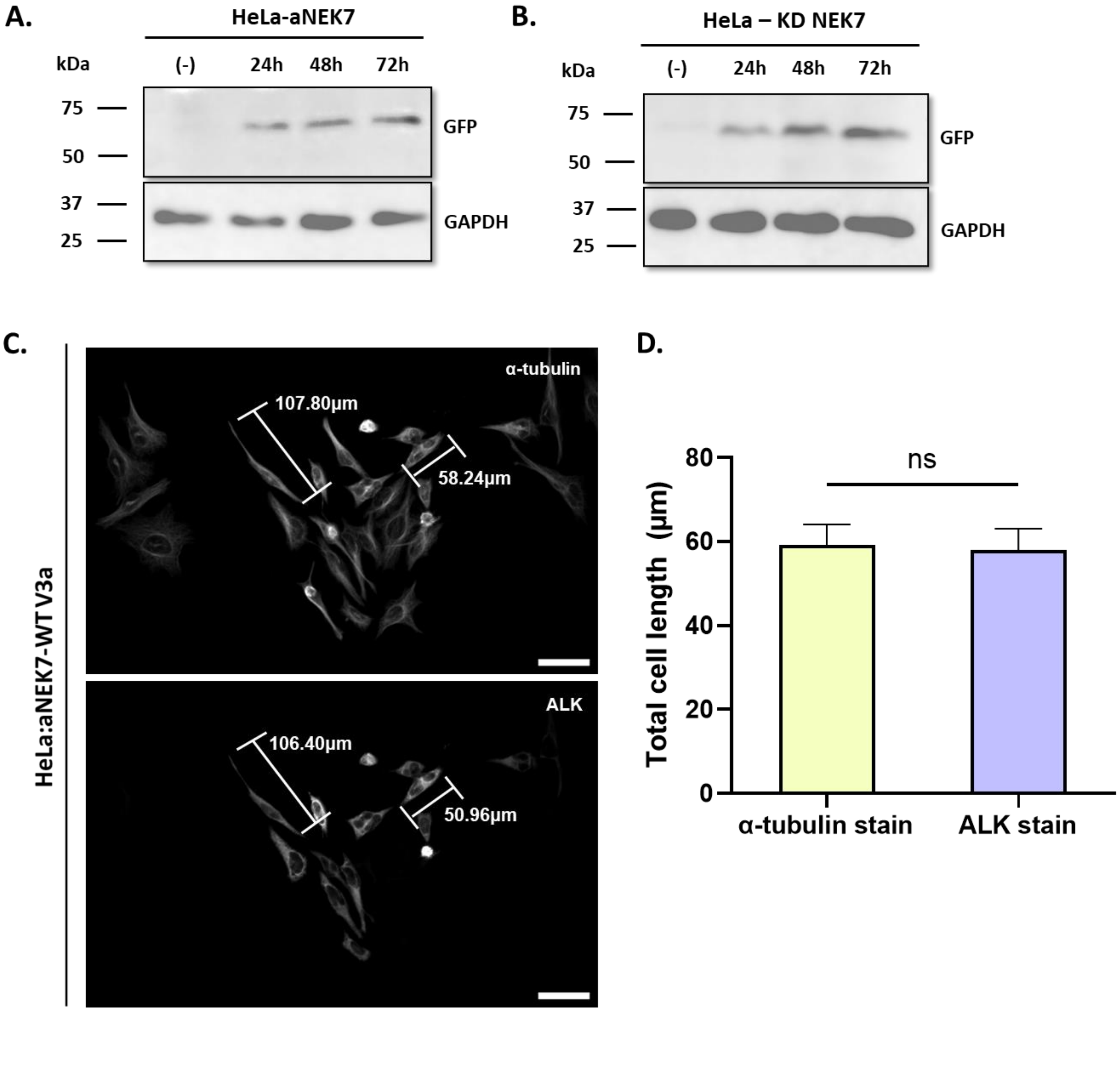
A.
HeLa-aNEK7
kDa
(-)
24h
48h
72h
75
50
GFP
37
25
GAPDH
B.
HeLa – KD NEK7
kDa
(-)
24h
48h
72h
75
50
GFP
37
25
GAPDH
C.
HeLa:aNEK7-WT V3a
α-tubulin
107.80μm
58.24μm
ALK
106.40μm
50.96μm
D.
Total cell length (μm)
80
60
40
20
0
ns
α-tubulin stain
ALK stain
