# Supplementary figures and images for: Microtubule Association of EML4–ALK V3 Is Key for the Elongated Cell Morphology and Enhanced Migration Observed in V3 Cells
Source: Cells. 2024 Nov 25;13(23):1954. doi: 10.3390/cells13231954 (PMC11639804; doi:10.3390/cells13231954)

## SUPPLEMENTARY DATA\_UNCROPPED GELS

Figure 1D-E

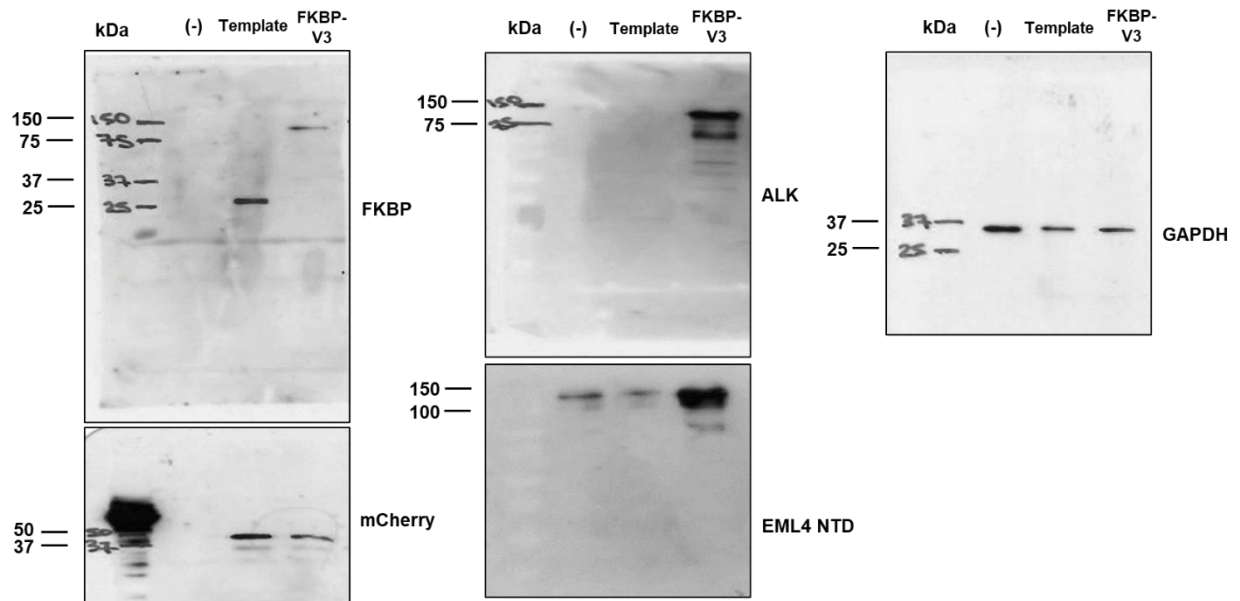

Figure 4H

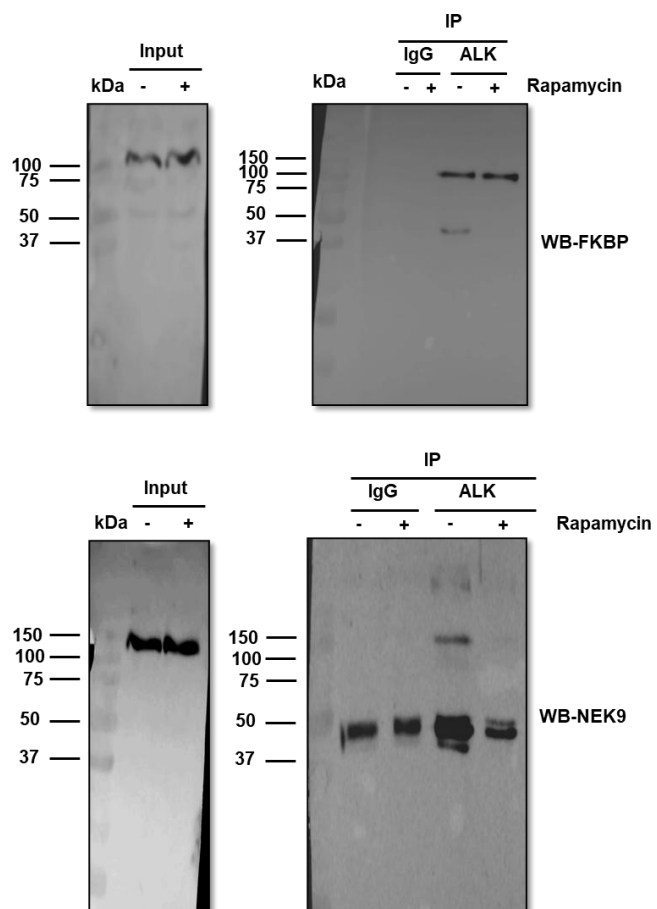

**Figure S1C**

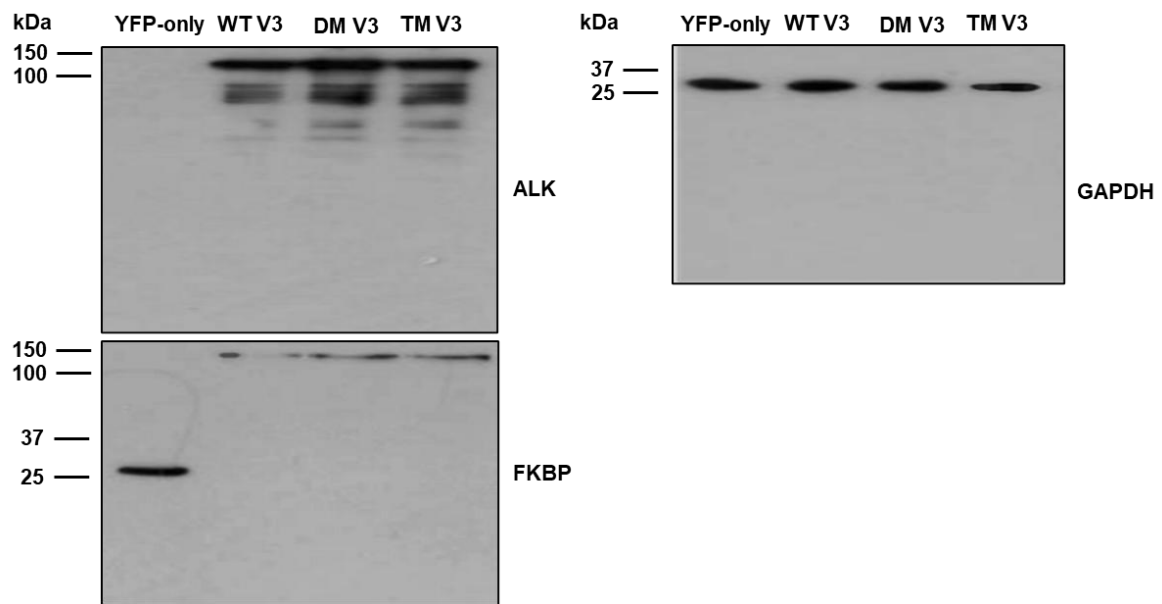

**Figure S2A**

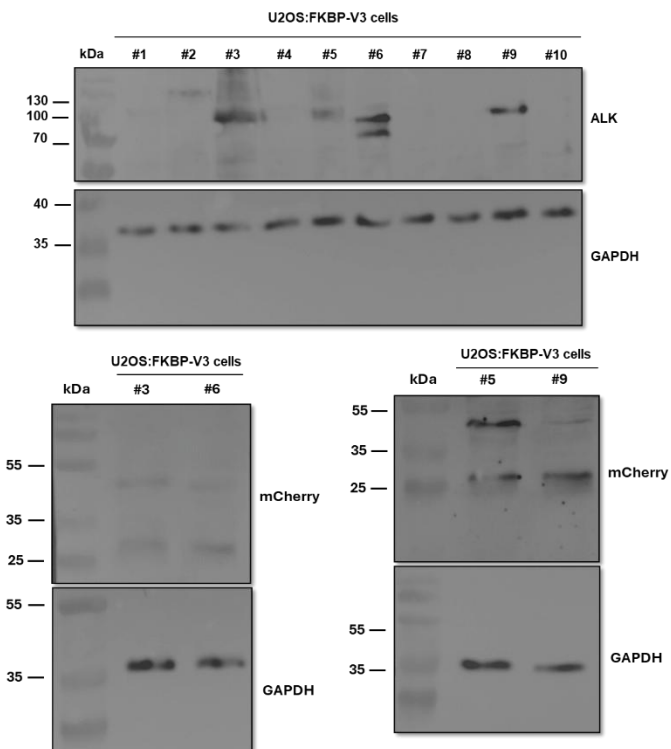

**Figure S3J**

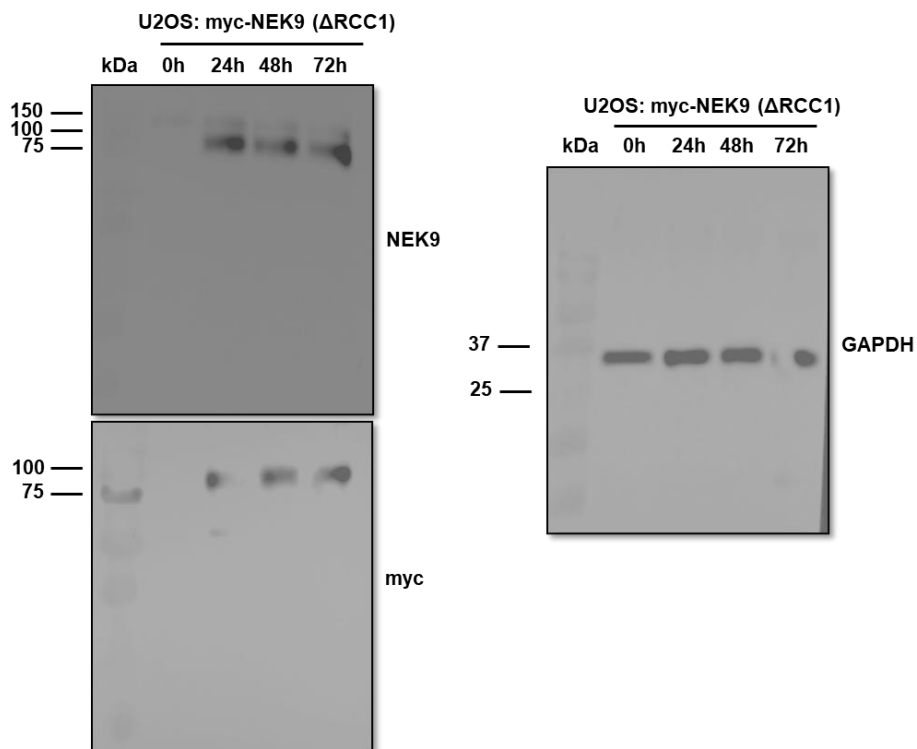

**Figure S4A-B**

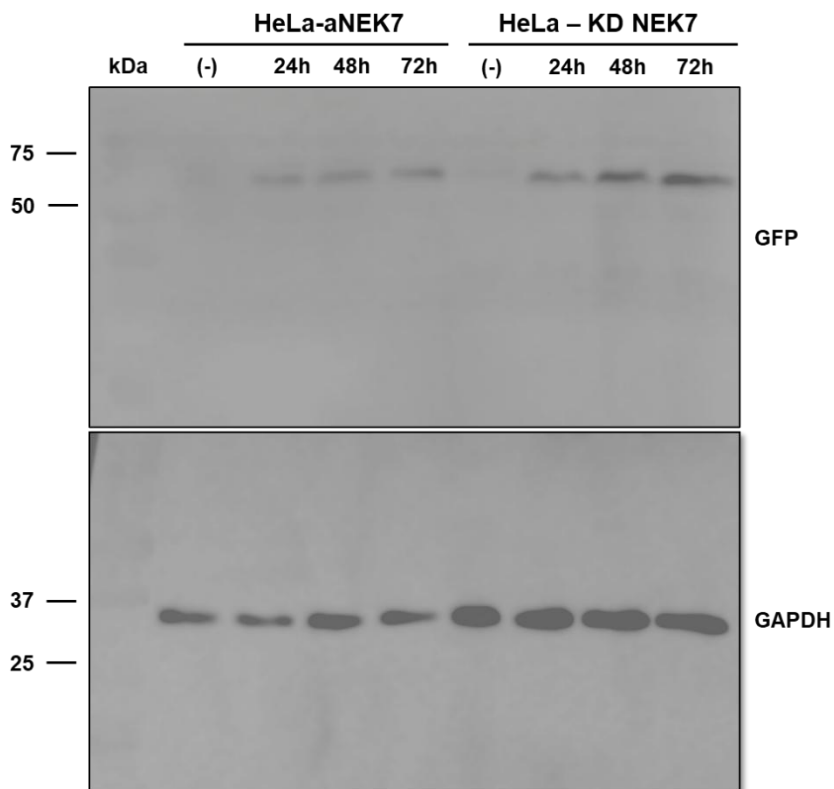

Supplement: Supplementary file 1 [file cells-13-01954-s001.zip › SUPPLEMENTARY DATA_UNCROPPED GELS_updated.pdf]
